# Comparison of the structure of floral nectaries in two *Euonymus* L. species (Celastraceae)

**DOI:** 10.1007/s00709-014-0729-6

**Published:** 2014-11-13

**Authors:** Agata Konarska

**Affiliations:** Department of Botany, Faculty of Horticulture and Landscape Architecture, University of Life Sciences in Lublin, Akademicka 15, 20-950 Lublin, Poland

**Keywords:** Celastraceae nectaries, *Euonymus*, Nectarostomata, Secretory epidermis cells, Phenolic compounds, Micromorphology and anatomy

## Abstract

The inconspicuous *Euonymus* L. flowers are equipped with open receptacular floral nectaries forming a quadrilateral green disc around the base of the superior ovary. The morphology and anatomy of the nectaries in *Euonymus fortunei* (Turcz.) Hand.-Mazz. and *Euonymus europaeus* L. flowers were analysed under a bright-field light microscope as well as stereoscopic and scanning electron microscopes. Photosynthetic nectaries devoid of the vascular tissue were found in both species. Nectar was exuded through typical nectarostomata (*E. fortunei*) or nectarostomata and secretory cell cuticle (*E. europaeus*). The nectaries of the examined species differed in their width and height, number of layers and thickness of secretory parenchyma, and the height of epidermal cells. Moreover, there were differences in the location and abundance of nectarostomata and the content of starch and phenolic compounds.

## Introduction

The family Celastraceae is represented mainly by trees, shrubs, and lianas inhabiting the tropical, subtropical, and moderate zones. In the previous classification system of angiosperms, the family Celastraceae was placed into the order Celastrales and it comprised three subfamilies: Celastroideae, Hippocrateoideae, and Salacioideae (Takhtajan 1980, 1997). In turn, according to the APG III system (APG 2009), three other families, i.e. Parnassiaceae, Lepuropetalaceae, and Pottingeriaceae have also been placed in Celastraceae. A representative of the subfamily Celastroideae is e.g. the genus *Euonymus* L. comprising 129 species whose distribution is concentrated in eastern Asia but they extend to Europe, northwest Africa, Madagascar, north and central America, and Australia (Ma 2001; Szweykowska and Szweykowski 2003). The inconspicuous protandrous *Euonymus* flowers arranged in apical umbellules are creamy-green. The actinomorphic tetramerous flowers are usually hermaphroditic, although secondary unisexuality of flowers, which is an effect of the reduction of either male or female reproductive organs of flowers, can also be found. The stamens alternate with petals, and the superior ovary base of the tetrahydronal pistil is surrounded by a well-developed annular nectariferous gland (Ding Hou 1962; Szweykowska and Szweykowski 2003; Thomas et al. 2011). The pollinators of *Euonymus* flowers include mainly Diptera and some Hymenoptera (Matthews and Endress 2005). According to Thomas et al. (2011), the flowers of *Euonymus europaeus* are nectar-rich and an important food source for hoverflies and other insects. Ants and beetles also visit Celastraceae flowers, perhaps to feed on pollen (Hilty 2014). Floral nectaries in different Celastraceae representatives occupy an area on the receptacle from the petals to the gynoecium or between the androecium and gynoecium; they may also be located between the androecium and petals annularly or with upturned margins. Sometimes they may form, with filament bases, a collar with broad interstaminal portions that have been interpreted as staminodes. Nectar is secreted through nectarostomata which are level with the epidermis, raised above, or sunken into pits (Sandvik and Totland 2003; Simmons 2004; Matthews and Endress 2005; Bernardello 2007; Gomes and Lombardi 2013).

The current knowledge of the structure of floral nectaries in the family Celastraceae concerns primarily the few representatives of Celastroideae and several neotropical species from the subfamily Salacioideae (Matthews and Endress 2005; Gomes and Lombardi 2013). With the exception of *Euonymus latifolius*, no information has been found about the function and structure of floral nectaries in other *Euonymus* species, which are relatively common representatives of the flora of the moderate climate zone. According to many authors, not only the position but also the structure of flower nectaries provide important taxonomic significance and can elucidate the origin and evolution of various plant groups (Percival 1961; Baker and Baker 1983; Endress 1995; Rudall et al. 2000, 2003; Smets et al. 2000; Bernardello 2007). Literature data indicate that within the family nectaries may be characterised by relatively great homogeneity (e.g. Rosaceae) or exhibit substantial diversity (e.g. Ranunculaceae) (Smets 1986; Erbar et al. 1998; Evans and Dickinson 2005; Bernardello 2007). The aim of the present paper is to check whether nectaries in the genus *Euonymus* retain a permanent model of the position and structure and whether their structure is a significant taxonomic trait for this genus, subfamily Celastroideae, and family Celastraceae. To this end, special focus was placed on the micromorphological and anatomical similarities and differences between the nectaries of two *Euonymus* species: *Euonymus fortunei* (Turcz.) Hand.-Mazz. and *Euonymus europaeus* L. Additionally, the mode of nectar production and release in these species was specified.

## Materials and methods

*Euonymus europaeus* and *E. fortunei* flowers at the stage of full bloom and nectar production (the second day after opening of petals) were collected in mid-May and mid-July 2013, respectively, in the UMCS Botanical Garden in Lublin. The structure of the nectary gland was analysed under the scanning electron microscope and light microscope (fresh material and fixed in 70 % ethanol).

### Scanning electron microscopy (SEM)

Five flowers from each *Euonymus* species were fixed in 4 % glutaraldehyde in 0.1 M phosphate buffer with a pH of 7.0. Next, the samples were dehydrated in an ethanol series and dried at the critical point in liquid CO_2_ (Bal-Tec CPD 030 critical point dryer) and coated with gold-palladium using the sputter coater EMITECH K550X. The preparations were observed under a TESCAN/VEGA LMU scanning electron microscope at an accelerating voltage of 30 kV. The length of nectarostomata (guard cells) was measured, and the number of stomata per square millimeter of the nectary epidermis was counted (*n* = 10) in each flower using the morphology software coupled with SEM.

### Light microscopy

Ten flowers from each *Euonymus* species were analysed under bright-field light (LM) and stereoscopic (SM) microscopes. The stereoscopic microscope was used for measurement of the flower diameter, length of petals, width and height of the nectary, and diameter of the ovary at the site surrounded by the nectary. Hand-made transverse and longitudinal sections of the flower with the nectary were prepared and viewed live; additionally, they were stained with IKI in order to detect starch and with FeCl_3_ to detect phenolic compounds (Johansen, 1940). Furthermore, in order to determine the function of abnormal cells present in the nectary epidermis of *E. europaeus*, additional histochemical assays were performed, including Sudan red B (Brundrett et al. 1991) and Sudan black B (Lison 1960) for lipids, Nile blue sulphate for acidic and neutral lipids (Cain 1947; Jensen 1962), and Nadi reagent for essentials oils detection (David and Carde 1964).

For examinations of the size and structure of the nectariferous glands, preparations from ten flowers of each *Euonymus* species were hand-made in glycerine jelly. Glandular parenchyma thickness in its mid-length, the height and width of epidermal cells were measured in the cross sections of the nectary tissue under a Nikon SE 102 light microscope; additionally, the number of the secretory parenchyma layers was counted.

### Statistical analyses

For all measured parameters, the means (±SD) were calculated. Data were analysed by one-way analysis of variance (ANOVA) and Tukey’s multiple range test for comparison of means, using software STATISTICA 7.0 (StatSoft, Inc., USA). The difference was considered statistically significant at the level of *P* < 0.05.

## Results

*Euonymus fortunei* and *E. europaeus* flowers are equipped with receptacular nectaries in the form of a dark green quadrilateral disc surrounding the base of the superior ovary. Nectar in the flowers of both species was visible after the petals opened, but the anthers were still closed.

**Fig. 1:**
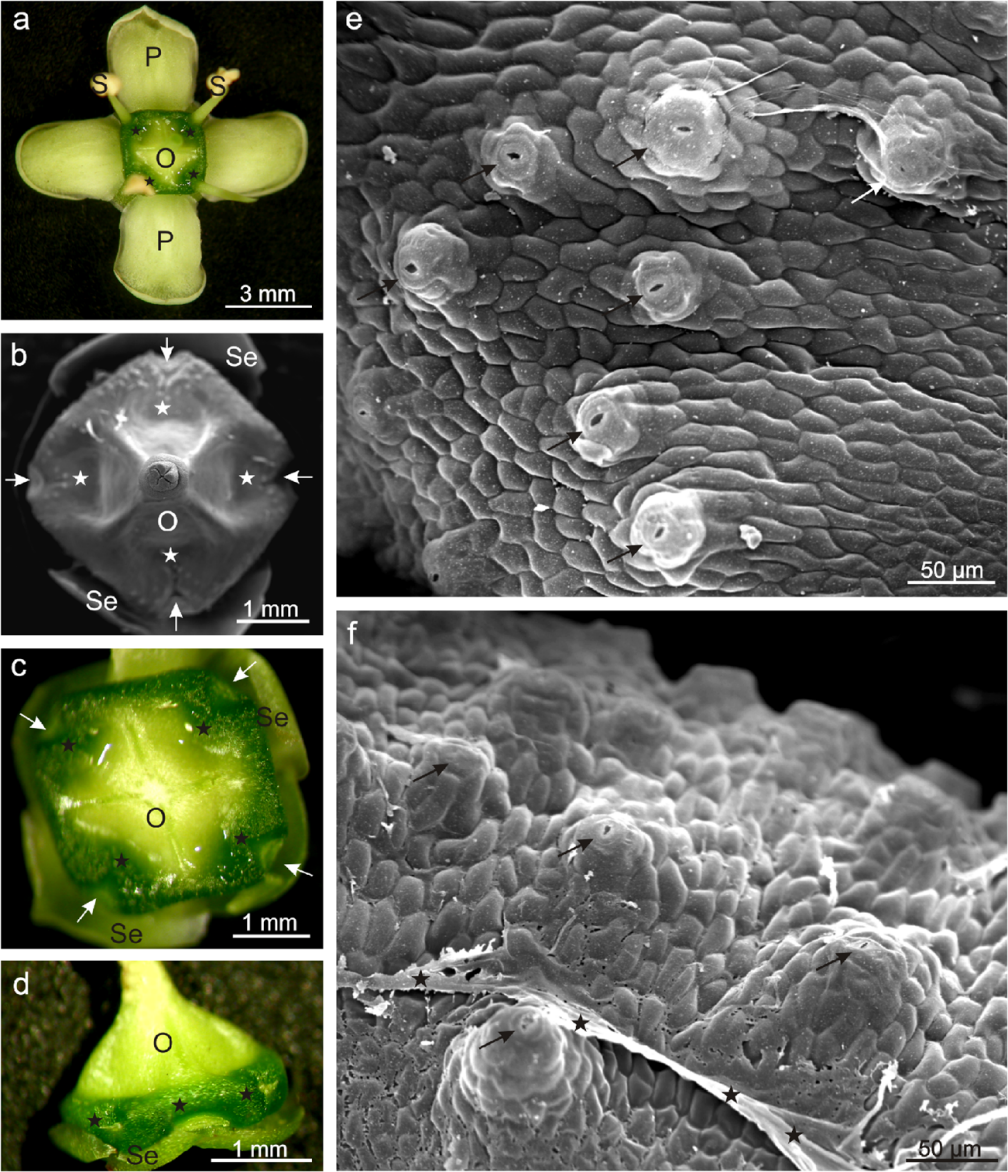
Morphology of *Euonymus fortunei* nectaries. **a** Flower with nectary (*asterisks*). **b**–**d** Nectaries with navicular cavities (*asterisks*) between the ovary and the site of stamen attachment (*arrows*). Note drops of nectar (**c**). **d** Lateral view; nectary disc around the ovary. **e**, **f** Nectary surface with nectarostomata (*arrows*) located on the convexities. Note secretions in the form of a protuberant layer (*asterisks*); *P* petals, *Se* sepals, *O* ovary, *S* stamens

The creamy-yellow *E. fortunei* flowers were approximately 1 cm in diameter, with the length of the petals accounting for approximately 60 %, and a discus with the tetrahydronal ovary covering the other part (Table 1, Fig. 1a). In *E. fortunei* flowers, the discus was located between the ovary and stamens attached around the perimeter of the nectary disc. The dark green colour of the nectary strongly contrasted with the creamy colour of the petals and the light green pistil (Fig. 1a–d). The width (top view) and height (lateral view) of the nectary disc were clearly differentiated in its perimeter. The parameters were the lowest between the stamens and the highest in the part opposite the stamens, where the characteristic cavities were found (Table 1, Fig. 1b–d).

**Table 1:** Characteristics of flowers and nectaries of *Euonymus fortunei* and *E. europaeus*

Parameters	*E. fortunei*	*E. europeaus*
Flower diameter (mm)	10 ± 0.9 a	8 ± 0.3 a
Petal length (mm)	3.1 ± 0.5 a	2.5 ± 1.2 a
Diameter of the nectary discus with the ovary (mm)	2.9 ± 0.5 a	2.6 ± 0.8 a
Diameter of the ovary (mm)	2.0 ± 0.2 a	1.2 ± 0.2 b
Nectary width (μm)	580 (310–905) ±48 a	660 (600–700) ±54 b
Nectary height (μm)	620 (500–710) ±66 a	256 (200–280) ±38 b
Number of nectarostomata (per mm^2^)	70 ± 3 a	210 ± 7 b
Length of the stomatal guard cell (μm)	25.4 ± 4.9 a	22.5 ± 5.6 a
Thickness of the glandular parenchyma layer (μm)	250 ± 13.7 a	148 ± 14.5 b
Number of glandular parenchyma layers	8–10 ± 2 a	4–5 ± 2 b
Height of epidermis cells (μm)	10.3 ± 2.4 a	30.4 ± 5.6 b
Width of epidermis cells (μm)	15.6 ± 5.7 a	16.6 ± 9.4 a
Presence of phenolic compounds	+++	+
Presence of starch grains	+	+++

Values are mean ± SD (standard deviation). Minimum and maximum values are given in brackets. Different letters within a line mean statistically significant differences (*P* < 0.05)

**Fig. 2:**
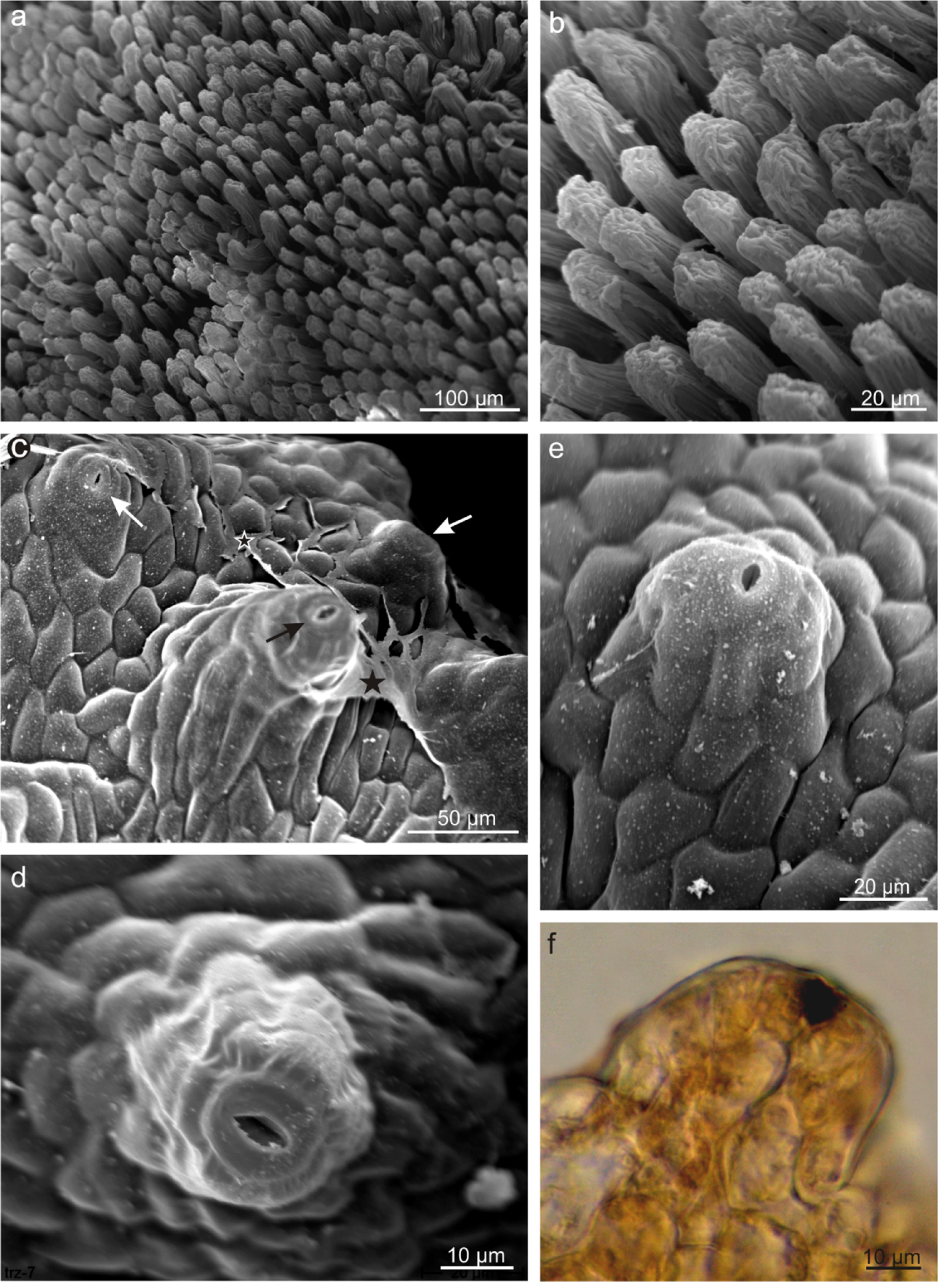
**a**, **b** Surfaces of *E. fortunei* petals with papillae and **c**–**f** flower nectaries with nectarostomata (*arrows*) located on the convexities. **b** Deep cuticular striae visible on the papillae surface. **c** A visible layer of dried secretion (*asterisks*)

Initially, nectar was accumulated in four navicular gland cavities (Fig. 1b, c), and next, it was able to leak out and stay on petals, whose surface was formed by numerous, high, obtuse papillae covered with an intensely striated cuticle (Fig. 2a, b). Nectar was exuded through numerous modified stomata, i.e. the so-called nectarostomata distributed uniformly on the upper surface of the entire gland as well as on its lateral parts (Table 1, Figs. 1e, f and 2c–f). Under SEM, a protuberant layer of dried secretion covered the nectariferous gland surface, especially in the vicinity of the stomata (Figs. 1e, f and 2c). The stomata were located on distinct convexities usually composed of 3–4 cell layers forming characteristic ca. 35-μm high ‘chimneys’ or ‘volcanoes’ (Figs. 1e, f and 2c–f).

**Fig. 3:**
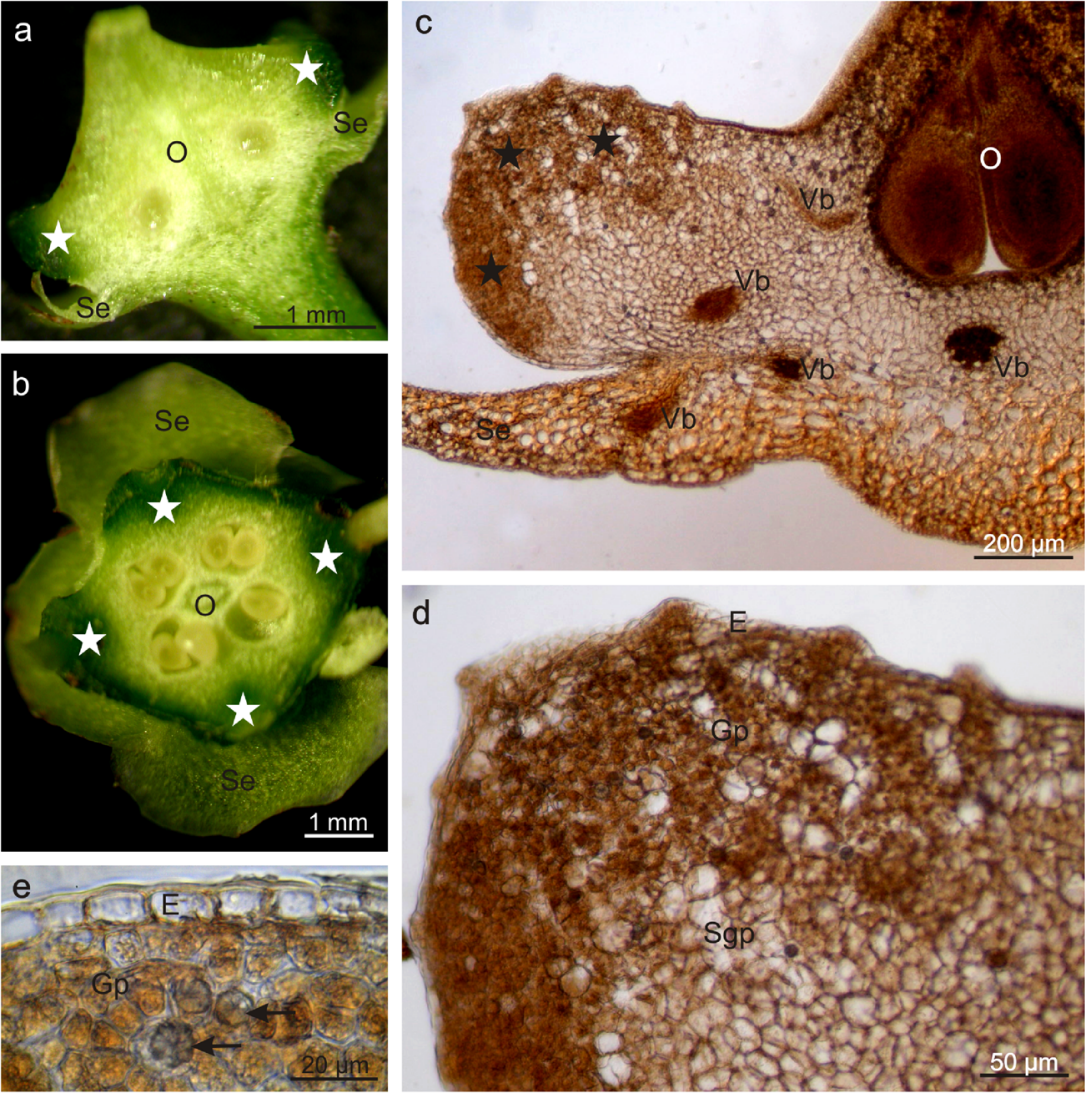
Anatomy of *E. fortunei* nectaries. **a**, **b** Varied thickness of the nectaries (*asterisks*) visible in the stereoscopic microscope. **a** Longitudinal section, **b** Cross section. **c**, **d** Longitudinal sections across the nectary. Note epidermis with nectarostomata and brown-coloured cells of glandular parenchyma containing phenolic compounds. **e** Druses (*arrows*) in the cells of the glandular parenchyma; *O* ovary, *Se* sepals, *Vb* vascular bundles, *E* epidermis, *Gp* glandular parenchyma, *Sgp* subglandular parenchyma

The longitudinal and transverse sections of the *E. fortunei* nectary revealed that the gland was formed by 8- to 10-layered glandular tissue and subglandular parenchyma (Table 1, Fig. 3a–e). Some glandular parenchyma cells contained chloroplasts, occasionally with starch grains, as well as calcium oxalate crystals and considerable numbers of orange-brown phenolic compound deposits (Fig. 3d–e), which turned brown-black when exposed to FeCl_3_ (not shown). The nectary surface comprised a single-layered epidermis, which was covered by a cuticle and contained stomata; the epidermal cells were rectangular in the cross section, and their width exceeded their height by approx. 50 % (Table 1, Fig. 3e). No vascularisation was detected in the secretory and subglandular parenchyma. However, vascular bundles of the sepals, receptacle, and ovary located nearby were noted (Fig. 3c).

**Fig. 4:**
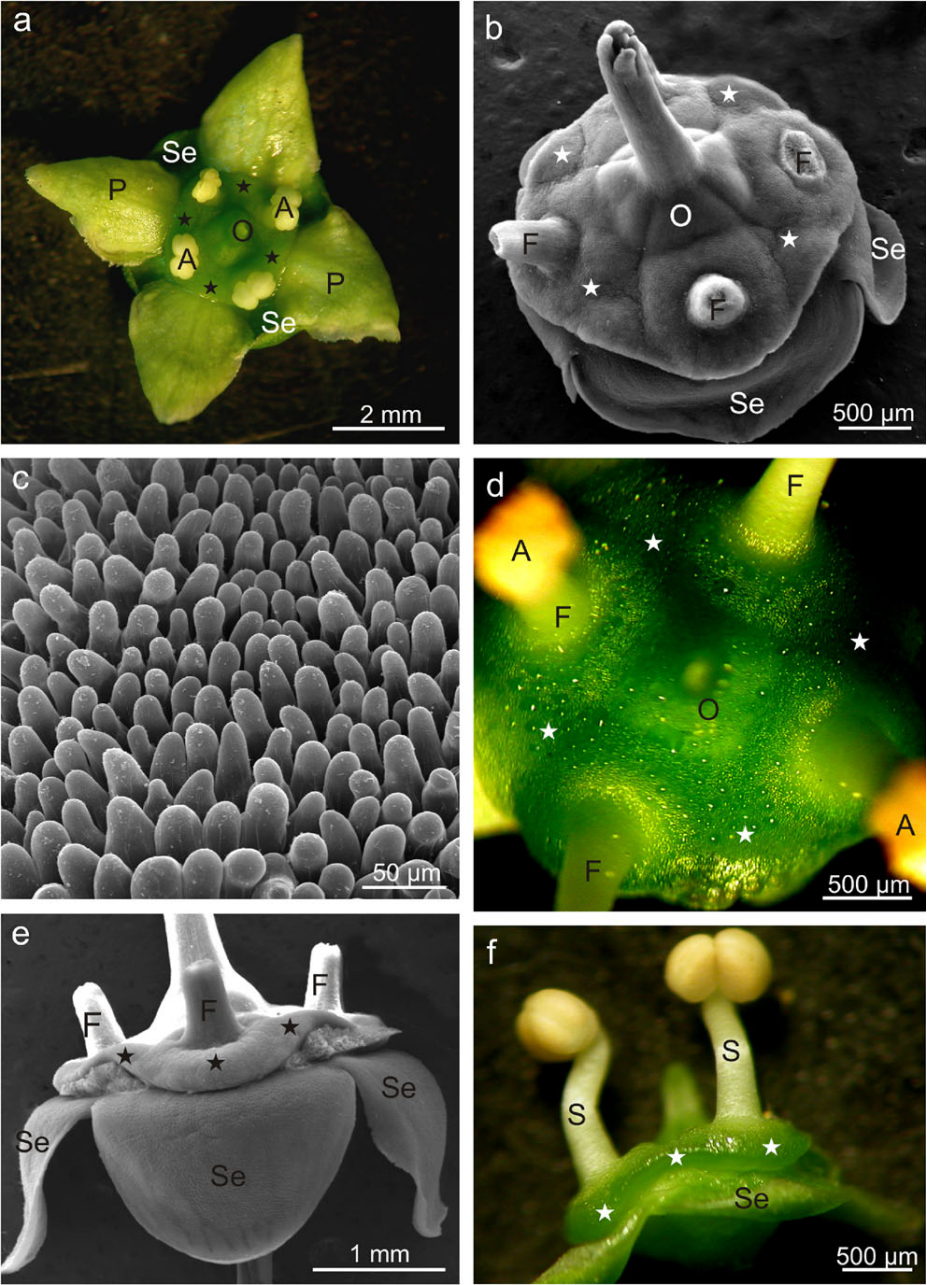
Morphology of *E. europaeus* flowers with nectaries (*asterisks*). **a** Flower with nectary with closed anthers. **b** Nectary in the form of a tetrahydronal disc with cavities surrounding the ovary. **c** Papillae on the flower petal. **d** Numerous nectar droplets visible on the nectary surface. **e**, **f** Varied thickness of the nectary visible in lateral view; *Se* sepals, *P* petals, *O* ovary, *S* stamens, *F* stamen filaments, *A* anthers

Compared with the *E. fortunei* flowers, the *Euonymus europaeus* flowers were smaller in diameter and in the length of petals. Additionally, the diameter of the ovary and the nectary width were lower by 44 and 11 %, respectively (Table 1, Fig. 4a, b). The nectary in *E. europaeus* occupied an area between the lower part of the ovary and petals. The stamens were attached within the discus, and the bases of their filaments were surrounded by an annular collar (Fig. 4a, b, d–f). Nectar secretion began when the anthers were still closed, and, at this stage, it proceeded exclusively through the nectarostomata in the epidermis of the lateral parts of the gland, whereas the upper part of the nectary disc was dry and devoid of secretion. After anther opening, numerous tiny nectar droplets were observed also on the upper surface of the gland both in the nectary cavities, which were shallower than those in *E. fortunei* and surrounded the ovary and filaments (Fig. 4b, d). Initially, nectar was accumulated in the space between the discus and receptacle, and later, it outflowed onto the petals. Some nectar remained on the petal surface thanks to the high and dense, nipple-shaped epidermal papillae covered with slightly striated cuticle (Fig. 4c), while another portion of the nectar flowed to the navicularly bent sepals. The width of the nectary disc measured from the ovary was similar along the gland perimeter (Table 1). In turn, the height of the *E. europaeus* nectary discus was lower than that of *E. fortunei*, but it was nonuniform along the perimeter (Table 1, Fig. 4e, f). Likewise in *E. fortunei*, the greatest nectary height was found between the stamens, and the lowest height was found for the cavities located between the stamens (Table 1, Fig. 4e, f).

**Fig. 5:**
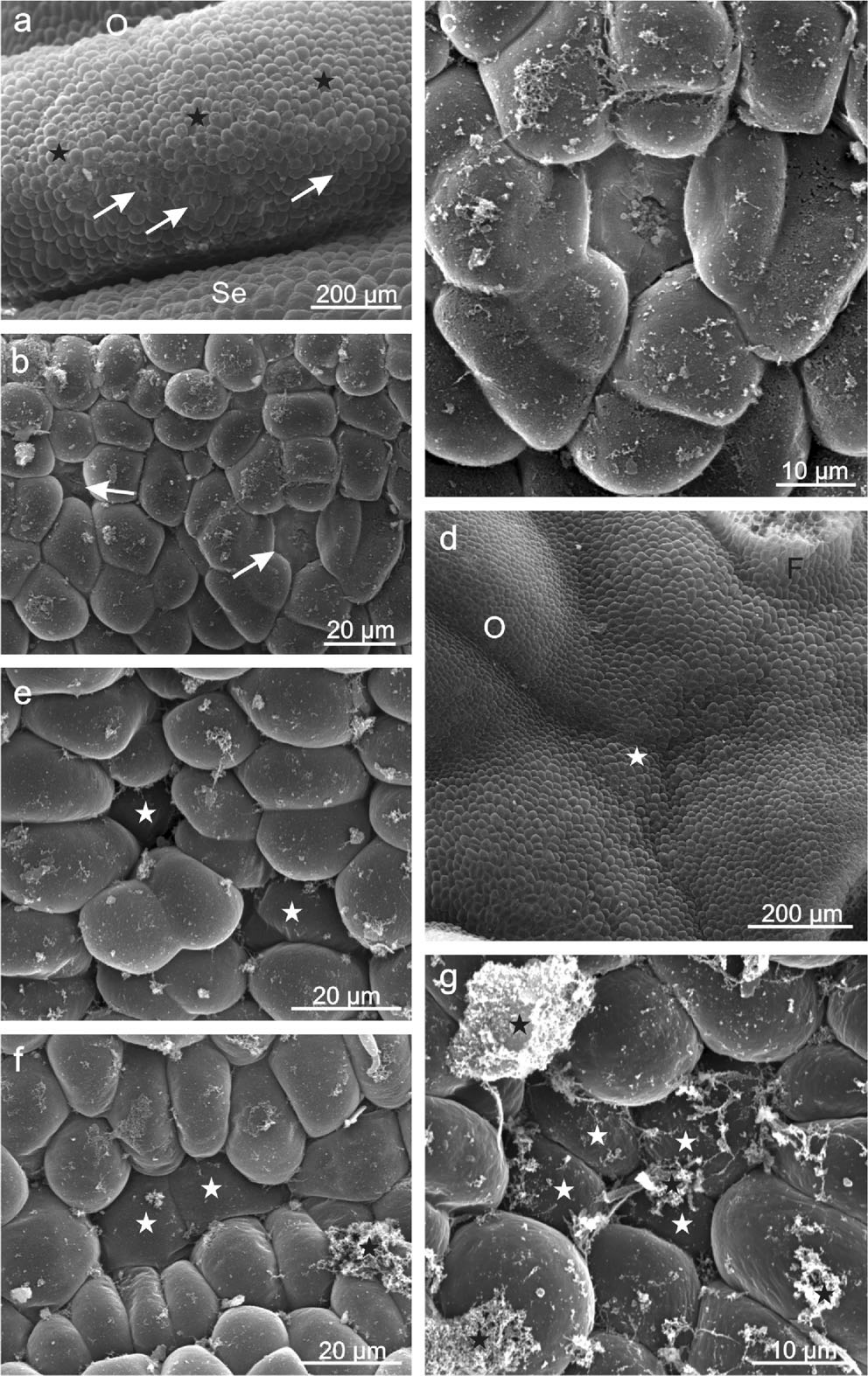
Surface of *E. europaeus* nectaries. **a** Fragment of the nectary (*asterisks*) with nectarostomata (*arrows*) (lateral view). **b** Nectarostomata on the lateral surface of the nectary (*arrows*). **c **Nectarostoma on the lateral surface of the nectary. **d** Fragment of the nectary surface with cavities (*asterisks*) between the ovary and the nectary margin (top view). **e**–**g** Planar and dark-coloured secretory cells (*white asterisks*) visible between typical epidermis cells with rounded outer cell walls. Note secretions in the form of the papules and floccules (*black asterisks*); *Se* sepal, *O* ovary, *F* stamen filaments

Nectar exudation in *E. europaeus* flowers proceeded in two ways depending on the part of the gland. In the epidermis of the lateral nectary parts, there were typical nectarostomata located below the level of the epidermal cells and arranged concentrically in several rows around the nectary discus (Fig. 5a–c). Their number was three-fold higher than that in *E. fortunei*, whereas their length was similar to that of *E. fortunei* nectarostomata (Table 1). In turn, atypical secretory cells were visible in the epidermis of the upper part of the discus, particularly in its cavities between typical epidermal cells with strongly convex external walls (Fig. 5d–g). These cells were arranged singly or in clusters and were characterised by flat external tangential walls, smaller sizes, and a darker colour under SEM; additionally, they were located below the level of adjacent epidermal cells (Fig. 5e–g). The surface of many of these secretory cells and neighbouring epidermal cells was often covered by abundant, dried secretion (Fig. 5g). Probably, the nectar penetrated apertures or channels in the cuticle of these cells, as no cracks or other damage were visible on their surface.

**Fig. 6:**
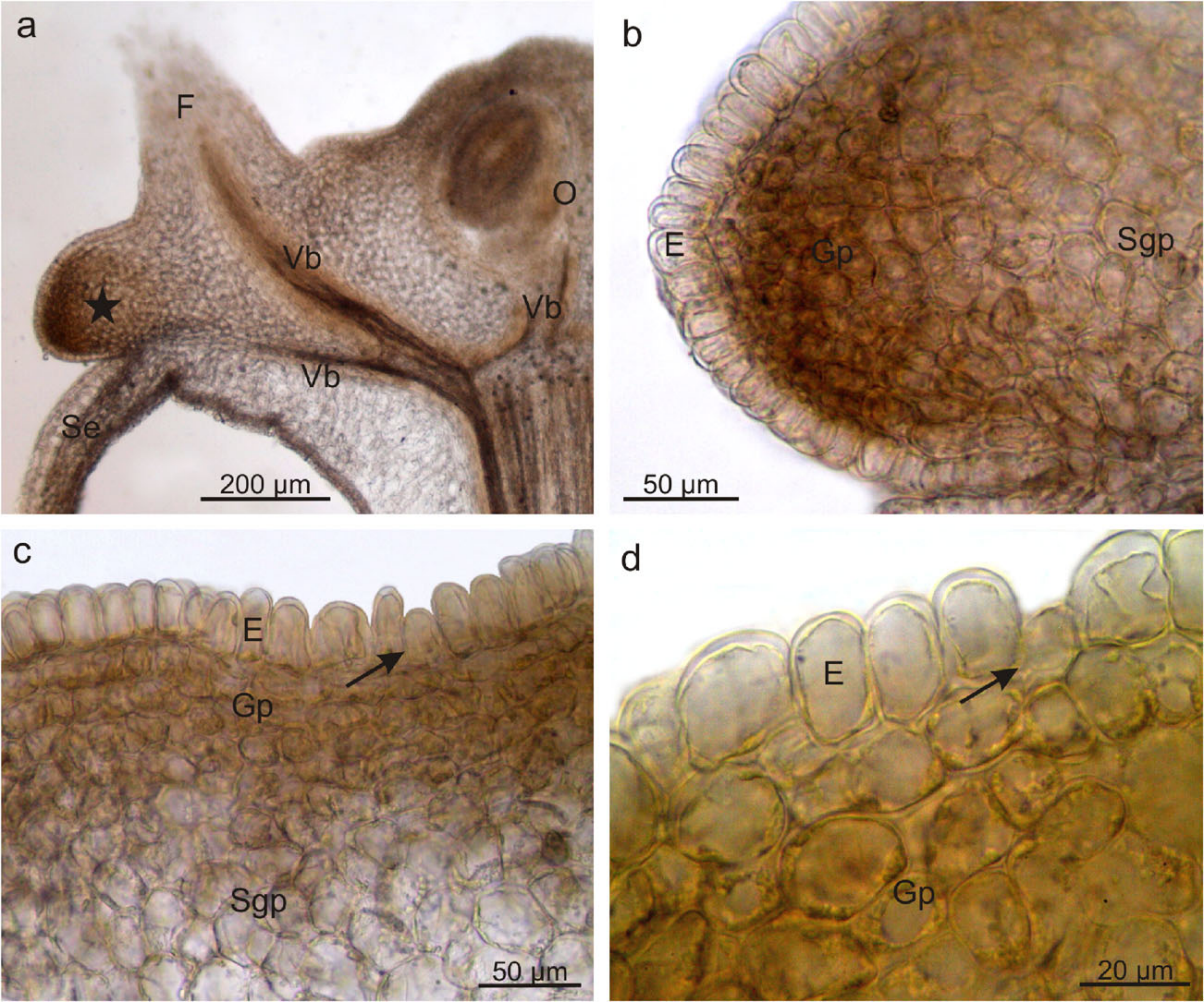
Longitudinal section through *E. europaeus* nectary. **a** Section through a flower with nectary (*asterisk*). **b**–**d** Fragments of sections through the nectary. Note the glandular parenchyma with a dark content of cells and epidermis with the rounded outer cell walls. **c**, **d** Lower secretory cells (*arrows*) visible in the epidermis; *Se* sepal, *O* ovary, *F* stamen filaments, *Vb* vascular bundles, *E* epidermis, *Gp* glandular parenchyma, *Sgp* subglandular parenchyma

**Fig. 7:**
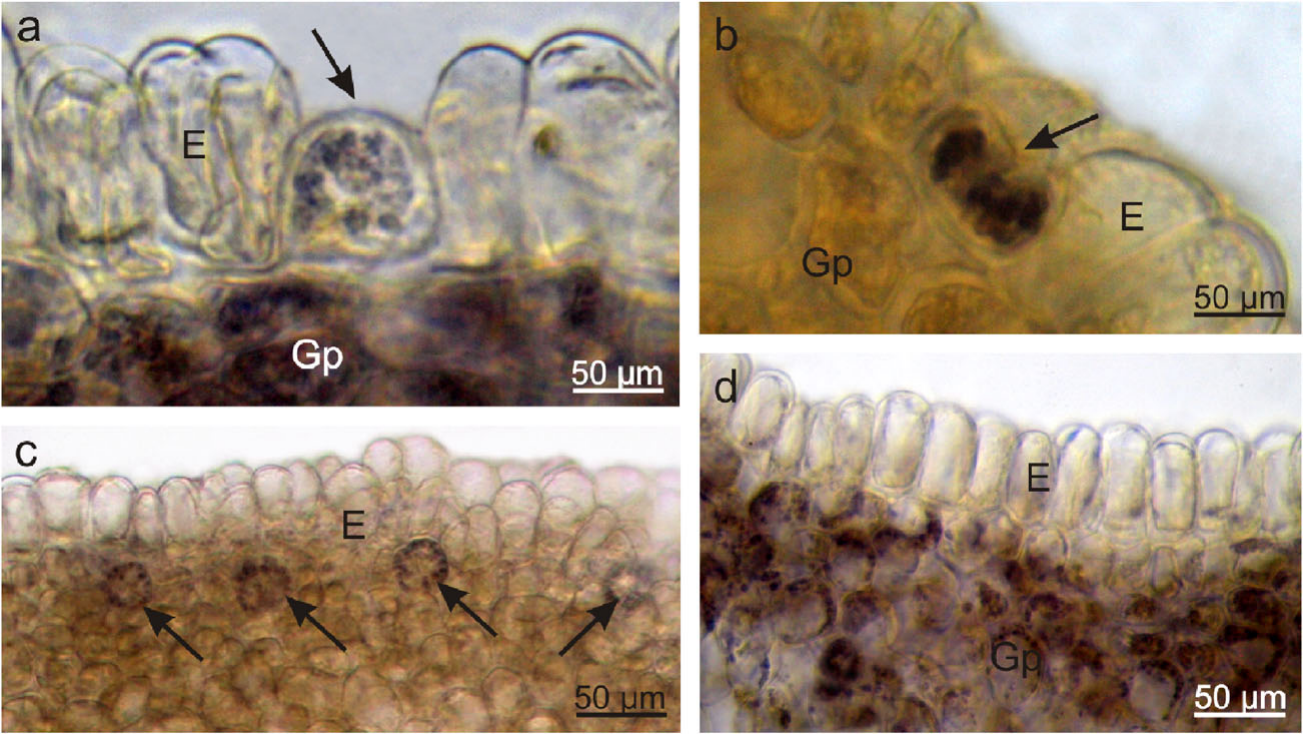
Fragments of the nectariferous tissue of *E. europaeus* treated with IKI. **a** Glandular parenchyma and a secretory cell (*arrow*) with almost black-stained starch grains. **b**, **c** Nectarostomata (*arrows*) in the nectary epidermis with stained starch grains. **d** Glandular parenchyma cells with stained starch grains; *E* epidermis, *Gp* glandular parenchyma

The longitudinal section of the *E. europaeus* nectary showed that the gland was composed of a single-layered epidermis and a multilayered glandular tissue devoid of vascularisation as well as subglandular tissue, close to which the vascular bundles of the sepals, ovary, and stamens were located (Fig. 6a–d). The *E. europaeus* nectariferous tissue was characterised by a nearly two-fold lower thickness and a two-fold lower number of layers than the secretory parenchyma in *E. fortunei* (Table 1). The epidermal cells, which were over two-folds higher than wider, had rounded external walls and a thin cuticle that was hardly visible under the light microscope (Fig. 6b–d). Their height was three-folds greater than that of the epidermal cells in *E. fortunei* nectaries (Table 1). Likewise SEM, longitudinal sections showed characteristic secretory cells between the nectary discus epidermal cells; they were characterised by a smaller height and lower content of intensely IKI-staining starch grains (Figs. 6c, d and 7a). Similar to other epidermal cells, these cells exhibited comparable response to the histochemical assays applied (not shown). In turn, the epidermis of the nectary lateral parts exhibited nectarostomata containing starch grains (Fig. 7b, c). In the cells of the glandular tissue, there were calcium oxalate crystals and numerous chloroamyloplasts filled with starch grains, whereas only few phenolic compound deposits were observed.

## Discussion

The floral nectaries in the analysed *Euonymus* species have a shape of a quadrilateral disc located on the receptacle and represent the type of open nectaries, which are easily accessible to insect pollinators. According to the classification of receptacular nectaries developed by Schmid (1988), which is based on the location of the gland relative to stamens, the *E. fortunei* nectary represents the intrastaminal type since it is located between the ovary and the androecium. In contrast, the gland in *E. europaeus* occupies an area extending from the ovary, between and around the stamens, to the perianth, i.e. it represents a combination of inter- and extrastaminal nectary types. Intrastaminal nectaries, in the form a flat platform around the gynoecium were observed also in *Euonymus latifolius* by Matthews and Endress (2005). Similarly located nectaries were also found in other Celastraceae representatives (Brasher 1998; Matthews and Endress 2005; Gomes and Lombardi 2013), with the exception of *Parnassia palustris*, which has staminal nectaries, and the role of the nectary is fulfilled by sterile stamens, the so-called staminodia (Sandvik and Totland 2003). Receptacular interstaminal nectariferous discs, which are intrastaminally flat and disciformous, but protuberant between the filament bases were also observed in representatives of the family Lepidobotryaceae from the order Celastrales (Link 1991).

The green *Euonymus* nectaries represent the type of photosynthetic mesenchymatous nectaries consisting of epidermis and glandular tissue. The glandular parenchyma contained chloro- or chloroamyloplasts, i.e. a site of synthesis of carbohydrates required for nectar production. Although the glandular tissue was not equipped with any type of vascular tissue, it seems that with the small size of *Euonymus* flowers and nectaries, the absence of vascularisation does not impair or inhibit nectar production. Furthermore, carbohydrates indispensable for nectar synthesis may also originate from the vascular bundles associated with the closely located sepal and/or pistil and/or receptacle traces. In *E. europaeus*, surplus carbohydrates were produced, which may be evidenced by the presence of starch in the secretory parenchyma cells. Similar observations of the mode of carbohydrate production and absence of vascularisation of the nectary were presented for several representatives of Lepidobotryaceae and Celastraceae by Link (1991) and Gomes and Lombardi (2013), whereas only phloematic bundles were observed in the nectaries of a few Celastroideae species by Matthews and Endress (2005), but not in *E. latifolius*. Nectaries devoid of their own vascular elements similar to those described for *Euonymus* have been reported from other species by many researchers (Fahn 1988, 2000; Galetto 1995; Ma et al. 2002; Ren et al. 2007; Konarska 2011). However, most frequently, floral nectaries comprise simultaneously xylem and phloem (Caswell and Davis 2011; Sulborska 2011; Abedini et al. 2013; Nores et al. 2013) or only phloem (Hampton et al. 2010; Zini et al. 2014).

Nectar exudation in the analysed *Euoynymus* species proceeds in two ways. In *E. fortunei*, it is achieved through nectarostomata located on nectary discus protuberances, whereas in *E. europaeus* through depressed nectarostomata (sunken below the epidermis cells), located only on the lateral surface of the gland and, probably, through microcracks or micropores in the thin cuticle of the characteristic epidermal secretory cells located on the dorsal surface of the nectary disc. Similar differentiation in the location of nectarostomata in relation to the level of epidermal cells in the nectaries of other Celastraceae species was described by Matthews and Endress (2005) and Gomes and Lombardi (2013). Furthermore, Matthews and Endress (2005) found that sunken nectarostomata in *E. latifolius* were located only on the upper surface of the nectary disc. Nectar exudation through nectarostomata is the most typical mode described in many species (Fahn 1988; Davis and Gunning 1993; Gaffal et al. 1998; Davis 2003; Abedini et al. 2013; Papp et al. 2013; Tobe 2013; Zini et al. 2014). In turn, atypical secretory cells, which occurred singly or in groups in the epidermis of the nectary disc in *E. europaeus*, were first described in Celastroideae. According to Gomes and Lombardi (2013), the nectar exudation process in Salacioideae may proceed through both the stomata and the nectary epidermal surface; additionally, the nectary in *Salacia elliptica* exhibited the characteristics of epithelial nectaries. Nectar release by orifices or small pores in the cuticle was first described by Vogel (1997). In various species, floral nectaries were described with nectar release through cuticle disruption, pores in cuticle, or rupture of the cell wall and cuticle (Figueiredo and Pais 1992; Vesprini et al. 1999, 2012; Weryszko-Chmielewska and Chwil 2007, Nocentini et al. 2012; Paiva 2012). Given the location and structure of the atypical secretory cells present in the epidermis of the *E. europaeus* nectary, the author has assumed that these may be underdeveloped stomata inhibited at an early stage of epidermis development. Inhibition of stomatal maturation on the upper nectary surface and the presence of functional stomata only on the lateral gland surface and additionally in the depressions contributes to limitation of nectar evaporation in this species. On the contrary, unlimited evaporation can occur through exposed and permanently opened stomata in *E. fortunei*. However, the number of stomata per unit area of the *E. fortunei* nectary was three-folds lower than that of *E. europaeus*, which may have compensated for the loss of nectar water in this species.

The cells of secretory parenchyma, mainly in *E. fortunei*, contained phenolic compounds. Their presence in the nectaries of other Celastraceae representatives has also been reported by Matthews and Endress (2005) and Gomes and Lombardi (2013), and phenolic compounds in the nectaries of other plant species have been described by other researchers (Beardsell et al. 1989; Espolador Leitão et al. 2005; De-Paula et al. 2011; Konarska 2013; Montenegro et al. 2013; Nepi 2014). Moreover, all the aforementioned *Euonymus* species organs and, particularly, fruits contain toxic glycosides and alkaloids applied in medicine (Thomas et al. 2011; Sharma et al. 2012; Zuo et al. 2012). The presence of secondary compounds such as phenolics, alkaloids, and terpenoids in nectary cells deter not only nectar-infecting microorganisms and foraging insects but also insect pollinators, e.g. bees (Adler 2000; Heil 2011). A similar role of a pest repellent may be attributed to druses, which are equally abundant in the nectary cells of both *Euonymus* species. The little interest in the flowers of the poisonous *Euonymus* plants exhibited by bees may be associated with the content of toxic compounds in the nectary cells and probably in the nectar itself. According to Baker and Baker (1982) and Adler (2000), the presence of toxic compounds in nectar is a characteristic feature of many poisonous plants. Moreover, Tan et al. (2007) argue that bees collect toxic nectar from poisonous plants only when the plants are the only source of nectar reward at a given time and place.

Nectaries in *Euonymus fortunei and E. europaeus* flowers exhibit a number of similarities (homogeneous traits) to nectaries described by other researchers in various representatives of Celastraceae. The similarities include the location of nectaries, mesenchymal type of nectaries, location and distribution of nectarostomata, absence of vascularization, and presence of phenolic compounds and druses. A unique (first time described) feature in the subfamily Celastroideae is the presence of epidermal secretory cells in *E. europaeus* nectaries. In turn, besides the homogeneous traits, there are distinct taxonomic differences between the nectaries of *E. fortunei* and *E. europaeus* mainly in terms of quantitative parameters, location, distribution, and abundance of nectarostomata, mode of nectar exudation, and content of phenolic compounds. The nectaries of the analysed *Euonymus* species and *E. latifolius* exhibit a number of not only common traits but also dissimilarities (Table 2).

**Table 2:** A comparison of flower nectaries in *E. fortunei*, *E. europaeus* (present studies), and *E. latifolius* (Matthews and Endress (2005))

Nectary features	*E. fortunei*	*E. europaeus*	*E. latifolius*
Form type	Prominent nectary disc on the receptacle		
	Intrastaminal	Mix of inter- and extrastaminal	Intrastaminal
Type of nectar exudation	Nectarostomata	Nectarostomata and secretory cell cuticle	Nectarostomata
Nectarostomata location	Raised	Sunken	Sunken
Nectarostomata distribution	Upper and lateral surface	Lateral surface	Upper surface
Epidermal secretory cells distribution	−	Upper surface	−
Vascular tissue	−	−	−
Phenolic compounds	+++	+	−
Oxalate druses	+	+	−
